# Associations between Fluid Intelligence and Physical Fitness in School Children

**DOI:** 10.3390/healthcare12100963

**Published:** 2024-05-08

**Authors:** Borja Bazalo, Verónica Morales-Sánchez, Nuria Pérez-Romero, Falonn Contreras-Osorio, Christian Campos-Jara, Antonio Hernández-Mendo, Rafael E. Reigal

**Affiliations:** 1 Faculty of Psychology, University of Malaga, 29016 Málaga, Spain; borjabd4@uma.es; 2 Department of Social Psychology, Social Anthropology, Social Work and Social Services, Faculty of Psychology, University of Malaga, 29016 Málaga, Spain; vomorales@uma.es (V.M.-S.); mendo@uma.es (A.H.-M.); 3 Exercise and Rehabilitation Sciences Institute, Postgraduate, Faculty of Rehabilitation Sciences, Universidad Andres Bello, Santiago de Chile 7591538, Chile; nuria.perez@unab.cl; 4 Exercise and Rehabilitation Sciences Institute, Faculty of Rehabilitation Sciences, Universidad Andres Bello, Santiago de Chile 7591538, Chile; falonn.contreras@unab.cl (F.C.-O.); christian.campos@unab.cl (C.C.-J.)

**Keywords:** fluid reasoning, fluid intelligence, physical activity, physical fitness, preadolescence, cognitive functioning

## Abstract

Previous research has highlighted that active lifestyles that contribute to improved physical fitness are positively related to cognitive functioning in children and adolescents. Specifically, the increase in physical condition at school age is considered relevant because it is related to better cognitive ability and greater academic performance. Thus, the aim of this study was to analyze the relationships between explosive strength, speed–agility, and fluid reasoning in schoolchildren. To achieve this objective, an associative, comparative, and predictive design was used in this research. A total of 129 children participated in this study (age: M = 9.48; SD = 0.99). To assess fluid reasoning, the Raven test’s Standard Progressive Matrices Subtest and the Wechsler Intelligence Scale for Children (WISC-V) were used. To assess physical fitness, the speed–agility test and the horizontal jump test (ALPHA-fitness battery tests), as well as the ball throw test (2 kg), were used. The results showed that the speed–agility test significantly predicted WISC-V Fluid Reasoning Index scores, and the medicine ball toss test significantly predicted Raven test scores. The results obtained highlight the associations between physical condition at these ages and fluid intelligence. This suggests that promoting active lifestyles that improve physical fitness could have a positive impact on children’s cognitive health.

## 1. Introduction

Pre-adolescence is a stage of multiple changes in which cognition plays a significant role in development [1,2]. Traditionally, the mental capacity responsible for reasoning, planning, and task resolution has been understood as intelligence, which has been further divided into two types: crystallized and fluid [3,4,5]. The former refers to the stored knowledge acquired throughout life, while fluid intelligence, also known as fluid reasoning, is defined as the ability to engage in thinking processes and solve problems in novel, unfamiliar, and unknown contexts [6,7]. Specifically, fluid reasoning plays a fundamental role in the educational context at these ages, as it is associated with working memory, inhibitory control, the ability to acquire knowledge swiftly, and problem-solving in complex environments, as well as psychosocial adaptation [8,9,10,11,12,13]. Thus, students with higher levels of fluid reasoning demonstrate superior academic performance, as they learn and consolidate new knowledge more efficiently [14].

Several studies have demonstrated the relationship between physical exercise and the development of cognitive skills, such as attention, memory, or inhibitory control, during this stage through various interventions. Some authors [15,16,17] have suggested that cognitive benefits are one of the consequences of the numerous neurophysiological changes that occur when engaging in regular physical exercise with a certain intensity. Therefore, it is important not only to determine the amount of activity school children engage in but also to assess their level of physical fitness [18,19]. In this regard, various studies have highlighted the existing relationship between physical fitness and cognitive functions at these ages [20,21,22]. For instance, a study conducted with Chilean students aged 9 to 12 [23] found better results in the gray and color trails test of the Neuropsychological Assessment of Executive Functions Battery for Children (ENFEN) in those students with higher scores in the 20 m shuttle run test, the standing broad jump (SBJ) test, and the 4 m × 10 m shuttle run test from the ALPHA-fitness battery. Another study with students aged 8 to 11 [24] also found associations between cognition and physical activity, assessed through Raven’s test and physical speed tests.

This relationship is justified by the changes that occur as a result of physical exercise in neuronal activity, such as increased blood flow, influencing the strength of synapses and favoring synaptic plasticity [25]. Additionally, it also leads to the release of growth factors and neurotransmitters associated with the improvement of physical fitness and synaptic plasticity [26]. Furthermore, some authors emphasize physical fitness as a determining factor in the effectiveness of sports practice for regulating brain-derived neurotrophic factor (BDNF), which aids the nervous system at a structural and functional level, improving communication between different cells, thereby facilitating learning and optimizing cognitive functions [15,27]. Thus, by strengthening the motor cognition network, which refers to the connections and brain circuits linking motor processing with cognitive processing, physical fitness may have a positive relationship with academic performance and cognitive functioning, as the brain regions involved in movement and coordination are also related to learning and other cognitive capacities [28,29].

Scientific research has explored the relationship between fluid reasoning and physical fitness in different population groups. For instance, Etnier and Berry [30] conducted a study on the effects of a 3-month exercise program and an 18-month exercise program on cognitive functioning in a sample of 40 individuals aged 55 to 80 years. The results showed that improvements in the dimensions of physical fitness, such as cardiorespiratory fitness, strength, and speed, were associated with improvements in fluid intelligence in both programs. In children and adolescents, Fochesatto et al. [31] conducted a study with 317 schoolchildren aged 6 to 11, analyzing the relationships between physical fitness and fluid intelligence. The results showed a statistically significant association between agility and fluid intelligence. However, there is limited literature studying these variables in pre-adolescence.

Most scientific studies have highlighted that aerobic capacity is the variable or dimension that best predicts cognitive functioning [32,33,34,35]. However, different authors have emphasized that other parameters of physical fitness, such as strength, speed, and agility, could be important for cognitive improvement. For example, Cherup et al. [36] observed the positive effects of a strength training program on executive functions. In turn, Chupel et al. [37] investigated the effect of strength training on cognitive functioning, observing improvements in various parameters. Nevertheless, most of these works have been conducted in older individuals, highlighting the need for more evidence in children and adolescents.

When examining the existing scientific literature, there are fewer studies addressing the relationship between cognitive functions and parameters of physical fitness other than aerobic capacity. Furthermore, research examining the relationship between fluid reasoning, speed–agility, and explosive strength in child and pre-adolescent populations is also limited. Studies that have analyzed the relationships between physical condition and cognitive functioning in children and adolescents have traditionally focused on aerobic capacity. However, it has been highlighted that other measures of physical fitness could explain part of these associations. To better understand this phenomenon, it is necessary to delve deeper into it and obtain data that allow us to understand the links to other dimensions of physical fitness. Therefore, given the importance of fluid intelligence in the development of children and adolescents, as well as the need to provide data on the relationship between physical capacities such as strength or speed with cognitive functioning, the present study is proposed. Thus, the aim of this study is to analyze the relationships between explosive strength and speed–agility with fluid reasoning in children aged 8 to 12. The research hypotheses are as follows: (a) statistically significant and positive relationships will be determined between explosive strength and speed–agility with fluid reasoning in children from 8 to 12 years old; (b) the predictive capacity of explosive strength and speed–agility on fluid reasoning in children from 8 to 12 years old will be determined.

## 2. Materials and Methods

### 2.1. Design

This research follows an associative, comparative, and predictive strategy [38].

### 2.2. Participants

A total of 129 boys and girls (males, n = 71; females, n = 58) from the city of Málaga (Spain) participated in this study, with ages ranging from 8 to 12 years (M = 9.48; SD = 0.99). All students were enrolled in the third to fifth grade of primary education. Non-probabilistic convenience sampling was employed. Inclusion criteria were as follows: (a) currently enrolled in third to fifth grade of primary school; (b) no muscular or osteoarticular injury preventing physical exercise. Exclusion criteria were as follows: (a) failure to provide informed consent, (b) having health problems or pathologies that hindered the completion of any test, (c) having a neuropsychological diagnosis that could affect the performance of cognitive tests, and (d) not attending the scheduled test date.

### 2.3. Instruments

(a) The Standard Progressive Matrices Subtest of Raven’s Progressive Matrices Test [39]: This test was designed to measure abstract reasoning ability and can be used from the age of 6. It consists of 60 matrices divided into five sets that increase in difficulty both within and between sets. Participants had to select one piece from 6 to 8 alternatives to complete each of the matrices containing geometric figures with a missing piece. The final score was obtained by summing the total correct answers from the five sets.

(b) The Matrices and Balances subtest of the Wechsler Intelligence Scale for Children (WISC-V) [40]: This test is designed to measure fluid intelligence and problem-solving ability in children and adolescents aged 6 to 16 years. Specifically, the matrices subtest consists of 32 matrices in which participants must select, from 5 alternatives, which piece fits into the missing space in each matrix. The matrices are presented in color and with increasing difficulty. The balance subtest consists of a total of 34 balances in which participants must choose, from 5 alternatives, the option that keeps the balance in equilibrium within a set time. To obtain the results of each test, it is necessary to convert the number of correct answers (raw score) into the scaled score assigned for each age group of participants. In our case, once this was achieved, it was necessary to calculate the Fluid Reasoning Index (FRI). To achieve this, the scaled scores of both tests were added together and compared in a conversion table to find the FRI.

(c) The ALPHA-fitness battery [41]: This battery was designed to provide a set of valid, safe, reliable, and feasible field tests for assessing physical fitness in children and adolescents. The horizontal jump test was selected to measure explosive lower body strength, and the 4 × 10 speed–agility test was chosen to measure speed, agility, and coordination. The horizontal jump test involved jumping forward from a static position. Participants, with their feet together at the jump line, performed a deep leg flexion and jumped to land as far away as possible. The horizontal distance between the line and the rearmost foot was measured in meters. The 4 × 10 speed–agility test was performed between two parallel lines separated by 10 m. Participants had to run from one line to the other four times as fast as possible, crossing the lines with both feet each time. Each time a participant crossed a line, they had to pick up a sponge and transfer it to the other side. The time taken was recorded in seconds. Both tests were individually evaluated for each participant.

(d) Explosive strength: The 2 kg medicine ball throw test [42] was implemented, which is used in the sports field to assess explosive strength and upper-body coordination. For its execution, participants positioned themselves on the throw line, keeping their feet immobile, and performed the throw using a trunk and arm flexion–extension. The distance from the throw line to the point where the ball touched the ground was recorded. It is worth noting that the test was evaluated individually for each participant.

### 2.4. Procedure

To carry out this research, contact was established with the school’s management, and permission was requested to conduct the research. Once approval was obtained, the research that was going to be developed was explained to the tutors and students of the school, and participation in the study was requested. Subsequently, informed consent was obtained from both the students and their parents/legal guardians. It was indicated that the data would be anonymous, participation was voluntary, and they could withdraw from the study at any time. Throughout the research process, the ethical principles of the Declaration of Helsinki [43] were respected. The study was approved by an ethics committee (CEUMA, nº 243, 19-2015-H) of the University of Málaga (Spain).

The cognitive functioning assessment tests were conducted in the school’s classrooms with those students who had authorized consent. Students who did not bring informed consent could not participate in the study. These students were in another classroom during the evaluations. The evaluations were in groups, and the students responded to the test in a self-reported way. They first took the Raven test and then the WISC tests, which were adapted to a digital format to be projected on a screen and administered in a group setting. The duration of both tests was approximately 90 min, with a 10 min break between tests. Two researchers administered the tests and were present during the tests to address any questions that arose. Subsequently, they performed the ALPHA-fitness battery and explosive strength tests. These tests were conducted on the school’s sports courts during school hours and on the same day for each group. The order followed was speed–agility, medicine ball throw, and horizontal jump. The physical condition evaluation tests were carried out individually, and the marks of each test were recorded by the researchers. The duration of the physical tests was approximately one hour. Both assessments were always conducted between 9:00 a.m. and 11:30 a.m. From the delivery of consent to obtaining the results, a total of 8 weeks passed, spanning the months of March, April, and May 2023.

Throughout the process, the evaluator and the teacher, who were knowledgeable about the tests, were present in each grade to address any questions that arose. They faced some setbacks, such as malfunctions in the digital screens used to project the templates, which were resolved by connecting a second device to them; another setback was the application time, which, although scheduled for one hour in each grade, was extended due to explanations, question resolutions, and template projection.

### 2.5. Data Analysis

Descriptive statistical analyses were conducted to evaluate the research data. The Kolmogorov–Smirnov test was used to analyze normality. Pearson’s coefficient was used to analyze the correlations between physical fitness and fluid intelligence, following the criteria established by Evans [44], to determine the size of the correlations: values between ±0.01 and ±0.19 indicate a very weak correlation, values between ±0.20 and ±0.39 indicate a weak correlation, values between ±0.40 and ±0.59 indicate a moderate correlation, and values between ±0.60 and ±0.79 indicate a high correlation. Linear regression analysis (stepwise) was performed to check the predictive capacity of the physical fitness tests on fluid intelligence. The significance level was set at α = 0.05. The SPSS Statistics software, V.23.0 (IBM, Inc., Chicago, IL, USA), was used for the statistical analysis.

## 3. Results

Table 1 displays the descriptive statistical analyses and the Kolmogorov–Smirnov normality test for the variables of the WISC-V, Raven, and physical fitness tests. To address normality issues with some variables that did not show a normal distribution, algorithms 1/x, x2, and ln(x) were applied, successfully normalizing data distributions in all cases except for Matrices (WISC-V).

Table 2 displays the correlations established between the variables of interest, fluid intelligence, and physical fitness. A statistically significant correlation was found between the speed–agility tests and the WISC-V variables (matrices (r = −0.20; *p* < 0.05), balances (r = −0.26; *p* < 0.01), composite score (r = −0.29; *p* < 0.01), Fluid Reasoning Index (r = −0.29; *p* < 0.01)), and the Standard Progressive Matrices Subtest of the Raven test (r = −0.26; *p* < 0.01). On the other hand, the jump test showed statistically significant correlations with the Standard Progressive Matrices Subtest of the Raven test (r = 0.24; *p* < 0.01) and an association close to significance with the following WISC-V variables: balances (r = 0.16; *p* = 0.06), composite score (r = 0.17; *p* = 0.054), and Fluid Reasoning Index (r = 0.17; *p* = 0.054). Finally, the throwing tests revealed a statistically significant correlation with the following WISC-V variables: balances (r = 0.20, *p* < 0.05), composite score (r = 0.19, *p* < 0.05), and Fluid Reasoning Index (r = 0.19, *p* < 0.05). A highly significant correlation was highlighted with the Standard Progressive Matrices Subtest of the Raven test (r = 0.34, *p* < 0.001), and a value close to significance was found with WISC-V matrices (r = −0.13, *p* = 0.08).

Table 3 presents the results of the stepwise linear regression analyses conducted to identify the physical fitness variables that predicted fluid reasoning values. The models met the assumptions of linearity in the relationship between predictor and criterion variables, homoscedasticity, and the normal distribution of residuals, with a mean value of zero and a standard deviation of one. The Durbin–Watson values were found to be between 2.18 and 2.31, which falls within the appropriate range. As established by Pardo and Ruiz [45], when the cutoff points are between 1.5 and 2.5, it can be assumed that the residuals are independent, satisfying the assumption of independence of the independent variables with respect to the dependent variable. Additionally, the tolerance (T) and variance inflation factor (VIF) values were adequate (1.00), considering that each model did not introduce more than one variable.

As observed, the analyses revealed that the speed–agility test significantly predicted the scores of the Fluid Reasoning Index (FRI) (WISC-V) (R = 0.29; Adjusted R^2^ = 0.08; F = 10.95; *p* < 0.001). Similarly, the throwing test significantly predicted the scores of the Standard Progressive Matrices Subtest of the Raven test (R = 0.34; Adjusted R^2^ = 0.11; F = 16.42; *p* < 0.001).

## 4. Discussion

The aim of this study was to analyze the relationships between upper-body explosive strength, speed–agility, and fluid reasoning in schoolchildren aged 8 to 12. The obtained results reveal statistically significant associations between the analyzed variables, fulfilling the research objective. These findings are in line with previous research that has highlighted the relationships between physical fitness and cognitive functions [23,24,46,47,48,49].

Firstly, correlation analyses revealed that participants with a higher level of physical fitness obtained statistically significant associations with the assessed fluid reasoning tests, both from the Standard Progressive Matrices Subtest of the Raven test and the WISC-V. These results align with previous research that has emphasized the relationship between physical fitness and better cognitive development at early ages [50,51,52]. Fochesatto et al. [31] conducted a study with 317 schoolchildren and found no association between cardiorespiratory and muscular fitness and fluid intelligence; however, Esteban-Cornejo et al. [53] did find a relationship between these variables. As discussed in Fochesatto et al. [31], these discrepancies may derive from the various assessments used (the 20 m shuttle run test and the maximal exercise test with a cycloergometer and intelligence with the Kaufman Brief Intelligence Test and Raven’s Colored Progressive Matrices). Additionally, the proposed physical fitness tests have not been extensively studied in this population, and there is limited research linking them to fluid intelligence. This represents an advancement in this field of knowledge and provides data regarding the relationship between physical fitness and cognition.

As previously described, the justification for these results could be situated in evidence that previously highlighted that the regular practice of physical activity at a certain intensity leads to changes in the structure and functionality of the brain, optimizing its development and contributing to improving brain plasticity [15,25,26,54]. These findings were also previously discussed in a review conducted by Ruiz-Ariza et al. [55]. This review looked at 10 studies examining the association between physical fitness and cognitive performance. It found that increasing the intensity of physical education classes, through interval aerobic running, has a positive influence on numerical speed and simple mathematical problem-solving. Although they also found that two out of three cross-sectional studies showed a positive relationship between physical fitness and cognitive performance, there was no clear consensus on this relationship. Although this research has a cross-sectional approach and causal conclusions cannot be drawn, it is plausible to hypothesize that participants with a higher level of physical fitness would have previously been subjected to higher levels of physical activity to achieve this level. Thus, those cognitive changes described in the literature would have gradually occurred, resulting in the findings found in this research. Therefore, possessing better physical fitness would be an indicator and a result of an active lifestyle, generating neurophysiological changes that would influence better cognitive development in the participants under study.

Secondly, the linear regression analysis reveals that speed–agility has statistically significantly predicted the Fluid Reasoning Index of the WISC-V, and the scores of the medicine ball throws have statistically significantly predicted the scores of the Standard Progressive Matrices Subtest of the Raven test. The obtained results are consistent with previous research that highlights statistically significant relationships between these variables. Moral-Campillo et al. [56] revealed an association between movement speed and cognitive processing speed. On the other hand, other conducted studies [57,58] have indicated that strength is related to a better ability to solve abstract problems. Aerobic fitness, linked to hippocampal volume, is related to performance in spatial and verbal learning. This suggests that physical fitness may influence fluid intelligence by promoting hippocampal changes and optimal cognitive functions in adolescents, offering a possible explanation for the observed results [59]. This would show that improving these physical capacities could have a positive impact on the parameters of fluid intelligence, emphasizing the importance of promoting programs that enhance these specific capacities to obtain benefits in the development of fluid intelligence in the early stages of life.

This study has several limitations. Firstly, the sample size is small and covers a limited number of grades. In future work, expanding the analyzed population could help establish more precise conclusions about this phenomenon. Secondly, the way the tests were administered was collective. Although aspects of brightness and noise were taken care of, this type of assessment could bias the performance of some students due to a loss of concentration or the pressure of being exposed to a group. On the other hand, this type of study can contribute to the association of variables, such as body mass composition, that could be related to fitness [60], so it could be interesting to continue investigating this type of variable and others through longitudinal analyses. Likewise, we recommend considering the inclusion of additional variables in future research, such as the sleep time of students, their nutritional status, and their academic level, to avoid potential biases in the obtained findings. Finally, it is important to consider that the time of day in which evaluations are carried out can generate a bias in results. This could be caused by individual predispositions and personal rhythms with respect to performing in both physical and cognitive tests.

However, the results obtained in this study contribute to expanding knowledge in an area that has been little explored and requires additional data to qualify previous evidence. Additionally, this study can be of great help to professionals and educational personnel who work directly with children and adolescents, as it provides valuable information for carrying out their work, promoting active lifestyles, and encouraging healthier development through physical activity. Thus, promoting active lifestyles plays a fundamental role in children’s development, and promoting more suitable habits at this stage can have a lasting impact on their lives [61].

## 5. Conclusions

The results of this research highlight the relationship between strength, speed–agility, and fluid intelligence. Fundamentally, statistically significant correlations were observed between speed–agility, throwing, and fluid intelligence. Furthermore, these physical fitness measures have shown predictive capacity in the fluid intelligence tests evaluated. Thus, the results of the present research indicate that both strength and speed–agility are dimensions of physical fitness that should be worked on to increase the development of fluid intelligence, which is very relevant for academic and personal functioning in children and adolescents. Therefore, in addition to relating aerobic capacity to the development of cognitive functioning, it would be interesting to assess other parameters of physical condition. To sum up, these findings suggest the importance of promoting the development of physical fitness for the improvement of cognitive functioning at these ages and provide data on the relevance of physical qualities other than aerobic capacity in establishing relationships with cognitive health.

## Figures and Tables

**Table 1:** Descriptive statistics and Kolmogorov–Smirnov test.

	***M***	***DT***	***S***	***K***	***K-S***
**WISC-V (Fluid Intelligence)**					
Matrices	9.13	2.65	−0.78	1.36	0.20 ***
Balances	9.17	3.41	−0.60	0.37	0.10 **
Composite Score	18.25	4.90	−0.68	0.18	0.10 **
Fluid Reasoning Index	94.97	14.32	−0.63	0.22	0.09 **
**Raven**					
Standard Progressive Matrices Subtest	33.90	7.39	−1.37	2.45	0.11 **
**Physical Fitness Test**					
Speed–Agility	13.65	1.33	0.92	2.20	0.10 **
Jump	1.57	0.26	−0.03	0.14	0.06
Throwing	3.29	0.92	0.93	0.74	0.08 *

Notes: M = mean; SD = standard deviation; S = skewness; K = kurtosis; K-S = Kolmogorov–Smirnov. * *p* < 0.05; ** *p* < 0.01; *** *p* < 0.001.

**Table 2:** Correlations between WISC-V variables, Raven’s Test Standard Progressive Matrices Subtest and physical fitness tests.

	Physical Fitness Test		
	*Speed–Agility*	*Jump*	*Throwing*
**WISC-V (Fluid Intelligence)**			
Matrices	−0.20 *	0.12	0.13 ^a^
Balances	−0.26 **	0.16 ^b^	0.20 *
Composite Score	−0.29 **	0.17 ^c^	0.19 *
Fluid Reasoning Index	−0.29 **	0.17 ^c^	0.19 *
**Raven**			
Standard Progressive Matrices Subtest	−0.26 **	0.24 **	0.34 ***

Notes: * *p* < 0.05; ** *p* < 0.01; *** *p* < 0.001; ^a^ *p* = 0.08; ^b^ *p* = 0.06; ^c^ *p* = 0.054.

**Table 3:** Linear regression analysis.

R	R^2^ Adjusted	D-W	Criterion	Predictors	Beta	*t*	*T*	*FIV*
0.29	0.08	2.18	FRI (WISC-V)	Speed–Agility	−0.29	−3.31 **	1.00	1.00
0.34	0.11	2.31	SPM Raven	Throwing	0.34	4.05 ***	1.00	1.00

Notes: R = correlation coefficient; R^2^ adjusted = adjusted coefficient of determination; D-W = Durbin–Watson; Beta = regression coefficient; T = tolerance; VIF = variance inflation factor; FRI = Fluid Reasoning Index; SPM = Standard Progressive Matrices Subtest. *** *p* < 0.001; ** *p* < 0.01.

## Data Availability

The data are available upon request from the authors.
